# 2,6-Bis[1-(2,4,6-tri­methyl­phenyl­imino)­eth­yl]pyridine

**DOI:** 10.1107/S1600536813033801

**Published:** 2013-12-18

**Authors:** Stuart M. Boyt, Adrian B. Chaplin

**Affiliations:** a Department of Chemistry, University of Warwick, Gibbet Hill Road, Coventry CV4 7AL, England

## Abstract

In the title mol­ecule, C_27_H_31_N_3_, the imine C=N groups are orientated *anti* to the pyridine N atom, with N—C—C—N torsion angles of −164.91 (11) and −170.53 (10)°. In the crystal, mol­ecules are connected by weak C—H⋯N and C—H⋯π inter­actions parallel to the *b* axis.

## Related literature   

For representative examples of the organometallic and catalytic chemistry of dimino­pyridine complexes, see: Britovsek *et al.* (1999 ▶); Dias *et al.* (2001 ▶); Liu *et al.* (2009 ▶); Wieder *et al.* (2011 ▶); Darmon *et al.* (2012 ▶). For the synthesis of 2,6-bis­[1-(2,4,6-tri­methyl­phenyl­imino)­eth­yl]pyridine, see: Britovsek *et al.* (1999 ▶).
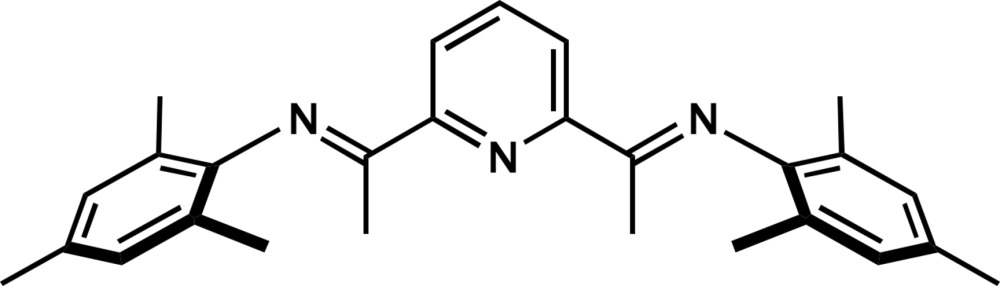


## Experimental   

### 

#### Crystal data  

C_27_H_31_N_3_
*M* *_r_* = 397.55
Triclinic, 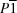

*a* = 8.2098 (3) Å
*b* = 11.4125 (4) Å
*c* = 13.0619 (4) Å
α = 79.224 (3)°
β = 77.066 (3)°
γ = 76.645 (3)°
*V* = 1148.65 (7) Å^3^
*Z* = 2
Cu *K*α radiation
μ = 0.52 mm^−1^
*T* = 150 K
0.5 × 0.4 × 0.3 mm

#### Data collection  

Oxford Diffraction Xcalibur (Ruby, Gemini) diffractometer
Absorption correction: multi-scan (*CrysAlis PRO*; Agilent, 2012 ▶) *T*_min_ = 0.679, *T*_max_ = 1.000
12720 measured reflections
4330 independent reflections
4028 reflections with *I* > 2σ(*I*)
*R*_int_ = 0.021

#### Refinement  

*R*[*F* ^2^ > 2σ(*F* ^2^)] = 0.044
*wR*(*F* ^2^) = 0.123
*S* = 1.04
4330 reflections
279 parameters
H-atom parameters constrained
Δρ_max_ = 0.22 e Å^−3^
Δρ_min_ = −0.20 e Å^−3^

### 

Data collection: *CrysAlis PRO* (Agilent, 2012 ▶); cell refinement: *CrysAlis PRO*; data reduction: *CrysAlis PRO*; program(s) used to solve structure: *SHELXS97* (Sheldrick, 2008 ▶); program(s) used to refine structure: *SHELXL97* (Sheldrick, 2008 ▶); molecular graphics: *Mercury* (Macrae *et al.*, 2008 ▶); software used to prepare material for publication: *OLEX2* (Dolomanov *et al.*, 2009 ▶).

## Supplementary Material

Crystal structure: contains datablock(s) I, global. DOI: 10.1107/S1600536813033801/tk5280sup1.cif

Structure factors: contains datablock(s) I. DOI: 10.1107/S1600536813033801/tk5280Isup2.hkl

Additional supporting information:  crystallographic information; 3D view; checkCIF report

## Figures and Tables

**Table 1:** Hydrogen-bond geometry (Å, °) *Cg*1 is the centroid of the (N1,C2–C6) ring.

*D*—H⋯*A*	*D*—H	H⋯*A*	*D*⋯*A*	*D*—H⋯*A*
C26—H26⋯N9^i^	0.95	2.64	3.5522 (18)	162
C28—H28*B*⋯*Cg*1^ii^	0.98	2.91	3.6715 (16)	135

Symmetry codes: (i) 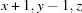; (ii) 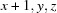.

**Fig. 1.**
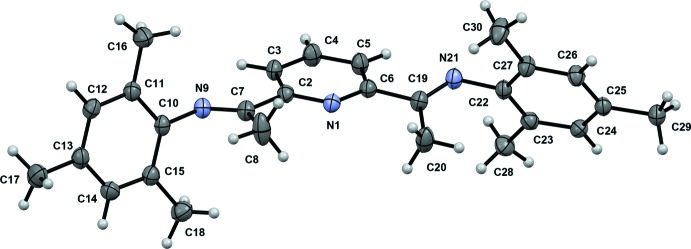
The molecular structure of (I); 50% probability displacement ellipsoids for the non-H atoms.

**Fig. 2.**
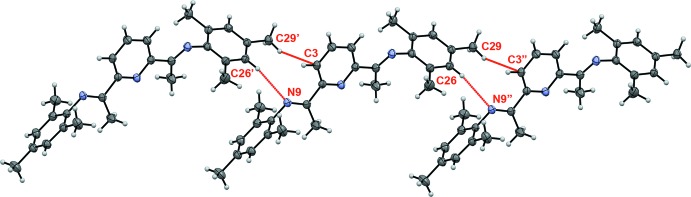
Weak intermolecular C—H···N and C—H···π interactions (highlighted in red) in the crystal of (I).

## supplementary crystallographic information

### 1. Comment

Ligands based on tridentate di­imino pyridines, containing bulky aryl substituents, find widespread application in organometallic chemistry and catalysis (Britovsek *et al.*, 1999; Dias *et al.*, 2001; Liu *et al.*, 2009; Wieder *et al.*, 2011; Darmon *et al.*, 2012). As part of our work using these ligands, we have determined the structure of the title compound (I).

Compound (I) adopts a conformation with the donor imine and pyridine groups orientated in opposing directions, with N—C—C—N dihedral angles of -164.91 (11)° and -170.53 (10)° (Fig. 1). This conformation contrasts with that observed on coordination of (I) to transitions metals (Britovsek *et al.*, 1999; Wieder *et al.*, 2011).

In the crystal the molecules are connected by weak C—H···N and C—H···π inter­actions, Table 1, parallel to the *b* axis (Fig. 2).

### 2.1. Synthesis and crystallization

The title compound (I) was prepared as previously described (Britovsek *et al.*, 1999). Crystallization from CH_2_Cl_2_—di­ethyl­ether at -20 °C afforded single crystals suitable for the crystallographic study.

### 2.2. Refinement

All aromatic-H atoms were placed in geometrically idealized positions (C—H = 0.95 Å) and constrained to ride on their parent atoms with *U*_iso_(H) = 1.2*U*_eq_(C). The methyl-H atoms were located in *SHELXL* with an ideal geometry (C—H = 0.98 Å and *U*_iso_(H) = 1.5*U*_eq_(C)).

### Crystal data

**Table:**

C_27_H_31_N_3_	*F*(000) = 428
*M**_r_* = 397.55	*D*_x_ = 1.149 Mg m^−^^3^
Triclinic, *P*1	Melting point: not measured K
*a* = 8.2098 (3) Å	Cu *K*α radiation, λ = 1.54184 Å
*b* = 11.4125 (4) Å	Cell parameters from 9488 reflections
*c* = 13.0619 (4) Å	θ = 3.5–78.0°
α = 79.224 (3)°	µ = 0.52 mm^−^^1^
β = 77.066 (3)°	*T* = 150 K
γ = 76.645 (3)°	Block, yellow
*V* = 1148.65 (7) Å^3^	0.5 × 0.4 × 0.3 mm
*Z* = 2	

### Data collection

**Table:**

Oxford Diffraction Xcalibur (Ruby, Gemini) diffractometer	4330 independent reflections
Radiation source: Enhance (Cu) X-ray Source	4028 reflections with *I* > 2σ(*I*)
Graphite monochromator	*R*_int_ = 0.021
Detector resolution: 10.2833 pixels mm^-1^	θ_max_ = 70.1°, θ_min_ = 7.0°
ω scans	*h* = −10→8
Absorption correction: multi-scan (*CrysAlis PRO*; Agilent, 2012)	*k* = −13→13
*T*_min_ = 0.679, *T*_max_ = 1.000	*l* = −15→15
12720 measured reflections	

### Refinement

**Table:**

Refinement on *F*^2^	Primary atom site location: structure-invariant direct methods
Least-squares matrix: full	Secondary atom site location: difference Fourier map
*R*[*F*^2^ > 2σ(*F*^2^)] = 0.044	Hydrogen site location: inferred from neighbouring sites
*wR*(*F*^2^) = 0.123	H-atom parameters constrained
*S* = 1.04	*w* = 1/[σ^2^(*F*_o_^2^) + (0.0686*P*)^2^ + 0.3065*P*] where *P* = (*F*_o_^2^ + 2*F*_c_^2^)/3
4330 reflections	(Δ/σ)_max_ = 0.001
279 parameters	Δρ_max_ = 0.22 e Å^−^^3^
0 restraints	Δρ_min_ = −0.20 e Å^−^^3^

### Special details

**Table:**

Geometry. All e.s.d.'s (except the e.s.d. in the dihedral angle between two l.s. planes) are estimated using the full covariance matrix. The cell e.s.d.'s are taken into account individually in the estimation of e.s.d.'s in distances, angles and torsion angles; correlations between e.s.d.'s in cell parameters are only used when they are defined by crystal symmetry. An approximate (isotropic) treatment of cell e.s.d.'s is used for estimating e.s.d.'s involving l.s. planes.

### Fractional atomic coordinates and isotropic or equivalent isotropic displacement parameters (Å^2^)

**Table:**

	*x*	*y*	*z*	*U*_iso_*/*U*_eq_	
N1	0.86531 (12)	0.40162 (9)	0.76760 (8)	0.0266 (2)	
N9	0.51220 (13)	0.63576 (9)	0.71925 (8)	0.0274 (2)	
N21	1.17638 (13)	0.18893 (9)	0.89040 (8)	0.0303 (2)	
C2	0.72569 (15)	0.48715 (10)	0.79075 (9)	0.0253 (3)	
C3	0.67074 (15)	0.52439 (11)	0.89048 (10)	0.0286 (3)	
H3	0.5721	0.5861	0.9042	0.034*	
C4	0.76288 (17)	0.46953 (12)	0.96906 (10)	0.0338 (3)	
H4	0.7280	0.4929	1.0380	0.041*	
C5	0.90604 (16)	0.38054 (12)	0.94679 (10)	0.0316 (3)	
H5	0.9706	0.3412	0.9999	0.038*	
C6	0.95368 (15)	0.34966 (10)	0.84446 (9)	0.0266 (3)	
C7	0.63022 (15)	0.54337 (11)	0.70241 (9)	0.0278 (3)	
C8	0.6824 (2)	0.48530 (15)	0.60287 (12)	0.0484 (4)	
H8A	0.6122	0.5304	0.5512	0.073*	
H8B	0.6664	0.4009	0.6195	0.073*	
H8C	0.8027	0.4867	0.5729	0.073*	
C10	0.41917 (15)	0.69900 (10)	0.63879 (9)	0.0264 (3)	
C11	0.25355 (15)	0.68233 (11)	0.64568 (10)	0.0290 (3)	
C12	0.16078 (15)	0.74925 (12)	0.56935 (10)	0.0310 (3)	
H12	0.0484	0.7379	0.5732	0.037*	
C13	0.22751 (16)	0.83187 (12)	0.48800 (10)	0.0320 (3)	
C14	0.39057 (16)	0.84921 (12)	0.48517 (10)	0.0325 (3)	
H14	0.4371	0.9066	0.4306	0.039*	
C15	0.48803 (16)	0.78501 (11)	0.55990 (10)	0.0298 (3)	
C16	0.17720 (18)	0.59491 (13)	0.73443 (12)	0.0415 (3)	
H16A	0.2493	0.5136	0.7331	0.062*	
H16B	0.0628	0.5923	0.7254	0.062*	
H16C	0.1697	0.6220	0.8026	0.062*	
C17	0.12676 (19)	0.90007 (15)	0.40411 (11)	0.0448 (4)	
H17A	0.1538	0.8543	0.3440	0.067*	
H17B	0.1565	0.9804	0.3798	0.067*	
H17C	0.0046	0.9098	0.4341	0.067*	
C18	0.66253 (17)	0.80796 (13)	0.55733 (11)	0.0381 (3)	
H18A	0.6678	0.8265	0.6267	0.057*	
H18B	0.6847	0.8770	0.5027	0.057*	
H18C	0.7486	0.7353	0.5409	0.057*	
C19	1.11231 (15)	0.25776 (11)	0.81485 (9)	0.0284 (3)	
C20	1.18436 (18)	0.25592 (14)	0.69860 (11)	0.0416 (3)	
H20A	1.2946	0.1993	0.6898	0.062*	
H20B	1.2000	0.3378	0.6646	0.062*	
H20C	1.1055	0.2295	0.6655	0.062*	
C22	1.32972 (15)	0.10129 (11)	0.86847 (9)	0.0272 (3)	
C23	1.48311 (16)	0.12957 (11)	0.87619 (9)	0.0275 (3)	
C24	1.63104 (15)	0.04141 (11)	0.86368 (9)	0.0274 (3)	
H24	1.7353	0.0600	0.8696	0.033*	
C25	1.63063 (15)	−0.07396 (11)	0.84263 (9)	0.0268 (3)	
C26	1.47697 (16)	−0.09938 (11)	0.83505 (10)	0.0295 (3)	
H26	1.4753	−0.1774	0.8202	0.035*	
C27	1.32457 (16)	−0.01400 (11)	0.84853 (10)	0.0307 (3)	
C28	1.48668 (18)	0.25374 (11)	0.89815 (11)	0.0364 (3)	
H28A	1.4610	0.3145	0.8372	0.055*	
H28B	1.6001	0.2545	0.9101	0.055*	
H28C	1.4013	0.2730	0.9614	0.055*	
C29	1.79323 (16)	−0.16854 (12)	0.83000 (10)	0.0326 (3)	
H29A	1.8755	−0.1391	0.7695	0.049*	
H29B	1.7687	−0.2443	0.8177	0.049*	
H29C	1.8411	−0.1836	0.8947	0.049*	
C30	1.15968 (18)	−0.04735 (14)	0.84300 (14)	0.0461 (4)	
H30A	1.0783	−0.0358	0.9095	0.069*	
H30B	1.1813	−0.1327	0.8322	0.069*	
H30C	1.1123	0.0048	0.7837	0.069*	

### Atomic displacement parameters (Å^2^)

**Table:**

	*U*^11^	*U*^22^	*U*^33^	*U*^12^	*U*^13^	*U*^23^
N1	0.0237 (5)	0.0260 (5)	0.0295 (5)	−0.0025 (4)	−0.0054 (4)	−0.0051 (4)
N9	0.0255 (5)	0.0265 (5)	0.0298 (5)	−0.0014 (4)	−0.0078 (4)	−0.0046 (4)
N21	0.0271 (5)	0.0291 (5)	0.0324 (5)	0.0015 (4)	−0.0064 (4)	−0.0062 (4)
C2	0.0238 (6)	0.0221 (5)	0.0301 (6)	−0.0038 (4)	−0.0058 (4)	−0.0039 (4)
C3	0.0254 (6)	0.0257 (6)	0.0325 (6)	0.0007 (5)	−0.0051 (5)	−0.0065 (5)
C4	0.0365 (7)	0.0347 (7)	0.0275 (6)	0.0023 (5)	−0.0065 (5)	−0.0088 (5)
C5	0.0316 (7)	0.0323 (6)	0.0291 (6)	0.0020 (5)	−0.0101 (5)	−0.0045 (5)
C6	0.0249 (6)	0.0246 (6)	0.0295 (6)	−0.0031 (5)	−0.0051 (5)	−0.0040 (4)
C7	0.0261 (6)	0.0268 (6)	0.0306 (6)	−0.0022 (5)	−0.0070 (5)	−0.0060 (5)
C8	0.0552 (9)	0.0479 (9)	0.0398 (8)	0.0190 (7)	−0.0235 (7)	−0.0196 (6)
C10	0.0263 (6)	0.0247 (6)	0.0273 (6)	0.0015 (4)	−0.0068 (5)	−0.0074 (4)
C11	0.0273 (6)	0.0268 (6)	0.0313 (6)	−0.0013 (5)	−0.0060 (5)	−0.0050 (5)
C12	0.0234 (6)	0.0351 (7)	0.0340 (6)	−0.0017 (5)	−0.0073 (5)	−0.0062 (5)
C13	0.0303 (6)	0.0344 (7)	0.0281 (6)	0.0024 (5)	−0.0076 (5)	−0.0049 (5)
C14	0.0330 (7)	0.0315 (6)	0.0289 (6)	−0.0033 (5)	−0.0032 (5)	−0.0011 (5)
C15	0.0270 (6)	0.0305 (6)	0.0304 (6)	−0.0021 (5)	−0.0042 (5)	−0.0070 (5)
C16	0.0330 (7)	0.0417 (8)	0.0468 (8)	−0.0096 (6)	−0.0113 (6)	0.0080 (6)
C17	0.0382 (8)	0.0556 (9)	0.0357 (7)	−0.0015 (7)	−0.0133 (6)	0.0039 (6)
C18	0.0328 (7)	0.0446 (8)	0.0371 (7)	−0.0108 (6)	−0.0061 (5)	−0.0035 (6)
C19	0.0253 (6)	0.0287 (6)	0.0310 (6)	−0.0019 (5)	−0.0063 (5)	−0.0067 (5)
C20	0.0362 (7)	0.0478 (8)	0.0323 (7)	0.0109 (6)	−0.0055 (5)	−0.0094 (6)
C22	0.0260 (6)	0.0264 (6)	0.0264 (6)	0.0010 (5)	−0.0054 (4)	−0.0036 (4)
C23	0.0305 (6)	0.0244 (6)	0.0265 (6)	−0.0049 (5)	−0.0042 (5)	−0.0029 (4)
C24	0.0248 (6)	0.0285 (6)	0.0286 (6)	−0.0061 (5)	−0.0056 (4)	−0.0020 (5)
C25	0.0264 (6)	0.0261 (6)	0.0255 (5)	−0.0010 (5)	−0.0052 (4)	−0.0025 (4)
C26	0.0308 (6)	0.0238 (6)	0.0345 (6)	−0.0025 (5)	−0.0082 (5)	−0.0073 (5)
C27	0.0268 (6)	0.0304 (6)	0.0359 (6)	−0.0027 (5)	−0.0089 (5)	−0.0073 (5)
C28	0.0367 (7)	0.0256 (6)	0.0469 (7)	−0.0059 (5)	−0.0066 (6)	−0.0071 (5)
C29	0.0282 (6)	0.0295 (6)	0.0377 (7)	0.0003 (5)	−0.0079 (5)	−0.0040 (5)
C30	0.0308 (7)	0.0409 (8)	0.0721 (10)	−0.0038 (6)	−0.0158 (7)	−0.0187 (7)

### Geometric parameters (Å, º)

**Table:**

N1—C2	1.3403 (15)	C16—H16C	0.9800
N1—C6	1.3382 (15)	C17—H17A	0.9800
N9—C7	1.2721 (15)	C17—H17B	0.9800
N9—C10	1.4247 (15)	C17—H17C	0.9800
N21—C19	1.2724 (16)	C18—H18A	0.9800
N21—C22	1.4252 (15)	C18—H18B	0.9800
C2—C3	1.3916 (17)	C18—H18C	0.9800
C2—C7	1.5011 (16)	C19—C20	1.5034 (17)
C3—H3	0.9500	C20—H20A	0.9800
C3—C4	1.3806 (17)	C20—H20B	0.9800
C4—H4	0.9500	C20—H20C	0.9800
C4—C5	1.3797 (17)	C22—C23	1.3989 (17)
C5—H5	0.9500	C22—C27	1.3999 (17)
C5—C6	1.3936 (17)	C23—C24	1.3864 (16)
C6—C19	1.4969 (16)	C23—C28	1.5055 (17)
C7—C8	1.5016 (17)	C24—H24	0.9500
C8—H8A	0.9800	C24—C25	1.3954 (17)
C8—H8B	0.9800	C25—C26	1.3861 (17)
C8—H8C	0.9800	C25—C29	1.5089 (16)
C10—C11	1.3969 (17)	C26—H26	0.9500
C10—C15	1.4009 (17)	C26—C27	1.3958 (17)
C11—C12	1.3943 (17)	C27—C30	1.5082 (18)
C11—C16	1.5045 (18)	C28—H28A	0.9800
C12—H12	0.9500	C28—H28B	0.9800
C12—C13	1.3862 (18)	C28—H28C	0.9800
C13—C14	1.3893 (19)	C29—H29A	0.9800
C13—C17	1.5099 (17)	C29—H29B	0.9800
C14—H14	0.9500	C29—H29C	0.9800
C14—C15	1.3934 (17)	C30—H30A	0.9800
C15—C18	1.5068 (18)	C30—H30B	0.9800
C16—H16A	0.9800	C30—H30C	0.9800
C16—H16B	0.9800		
			
C6—N1—C2	118.11 (10)	H17A—C17—H17B	109.5
C7—N9—C10	121.06 (10)	H17A—C17—H17C	109.5
C19—N21—C22	120.38 (10)	H17B—C17—H17C	109.5
N1—C2—C3	122.74 (11)	C15—C18—H18A	109.5
N1—C2—C7	116.51 (10)	C15—C18—H18B	109.5
C3—C2—C7	120.75 (10)	C15—C18—H18C	109.5
C2—C3—H3	120.8	H18A—C18—H18B	109.5
C4—C3—C2	118.45 (11)	H18A—C18—H18C	109.5
C4—C3—H3	120.8	H18B—C18—H18C	109.5
C3—C4—H4	120.2	N21—C19—C6	117.14 (11)
C5—C4—C3	119.53 (11)	N21—C19—C20	125.45 (11)
C5—C4—H4	120.2	C6—C19—C20	117.39 (10)
C4—C5—H5	120.8	C19—C20—H20A	109.5
C4—C5—C6	118.41 (11)	C19—C20—H20B	109.5
C6—C5—H5	120.8	C19—C20—H20C	109.5
N1—C6—C5	122.75 (11)	H20A—C20—H20B	109.5
N1—C6—C19	116.71 (10)	H20A—C20—H20C	109.5
C5—C6—C19	120.51 (11)	H20B—C20—H20C	109.5
N9—C7—C2	116.71 (10)	C23—C22—N21	118.40 (11)
N9—C7—C8	126.19 (11)	C23—C22—C27	120.87 (11)
C2—C7—C8	117.11 (10)	C27—C22—N21	120.51 (11)
C7—C8—H8A	109.5	C22—C23—C28	120.32 (11)
C7—C8—H8B	109.5	C24—C23—C22	118.95 (11)
C7—C8—H8C	109.5	C24—C23—C28	120.73 (11)
H8A—C8—H8B	109.5	C23—C24—H24	119.2
H8A—C8—H8C	109.5	C23—C24—C25	121.62 (11)
H8B—C8—H8C	109.5	C25—C24—H24	119.2
C11—C10—N9	119.09 (11)	C24—C25—C29	120.58 (11)
C11—C10—C15	120.84 (11)	C26—C25—C24	118.22 (11)
C15—C10—N9	119.80 (11)	C26—C25—C29	121.20 (11)
C10—C11—C16	120.41 (11)	C25—C26—H26	118.9
C12—C11—C10	118.55 (11)	C25—C26—C27	122.12 (11)
C12—C11—C16	121.04 (11)	C27—C26—H26	118.9
C11—C12—H12	119.0	C22—C27—C30	121.65 (11)
C13—C12—C11	122.01 (12)	C26—C27—C22	118.21 (11)
C13—C12—H12	119.0	C26—C27—C30	120.13 (11)
C12—C13—C14	118.10 (11)	C23—C28—H28A	109.5
C12—C13—C17	121.00 (12)	C23—C28—H28B	109.5
C14—C13—C17	120.90 (12)	C23—C28—H28C	109.5
C13—C14—H14	119.0	H28A—C28—H28B	109.5
C13—C14—C15	122.05 (11)	H28A—C28—H28C	109.5
C15—C14—H14	119.0	H28B—C28—H28C	109.5
C10—C15—C18	120.44 (11)	C25—C29—H29A	109.5
C14—C15—C10	118.38 (11)	C25—C29—H29B	109.5
C14—C15—C18	121.18 (11)	C25—C29—H29C	109.5
C11—C16—H16A	109.5	H29A—C29—H29B	109.5
C11—C16—H16B	109.5	H29A—C29—H29C	109.5
C11—C16—H16C	109.5	H29B—C29—H29C	109.5
H16A—C16—H16B	109.5	C27—C30—H30A	109.5
H16A—C16—H16C	109.5	C27—C30—H30B	109.5
H16B—C16—H16C	109.5	C27—C30—H30C	109.5
C13—C17—H17A	109.5	H30A—C30—H30B	109.5
C13—C17—H17B	109.5	H30A—C30—H30C	109.5
C13—C17—H17C	109.5	H30B—C30—H30C	109.5

### Hydrogen-bond geometry (Å, º)

Cg1 is the centroid of the (N1,C2–C6) ring.

**Table:**

*D*—H···*A*	*D*—H	H···*A*	*D*···*A*	*D*—H···*A*
C26—H26···N9^i^	0.95	2.64	3.5522 (18)	162
C28—H28*B*···*Cg*1^ii^	0.98	2.91	3.6715 (16)	135

Symmetry codes: (i) *x*+1, *y*−1, *z*; (ii) *x*+1, *y*, *z*.
